# Biomimetic Nanoparticles Camouflaged in Cancer Cell Membranes and Their Applications in Cancer Theranostics

**DOI:** 10.3389/fonc.2019.01560

**Published:** 2020-01-21

**Authors:** Jiefu Jin, Zaver M. Bhujwalla

**Affiliations:** ^1^Division of Cancer Imaging Research, The Russell H. Morgan Department of Radiology and Radiological Science, Baltimore, MD, United States; ^2^Sidney Kimmel Comprehensive Cancer Center, Baltimore, MD, United States; ^3^Department of Radiation Oncology and Molecular Radiation Sciences, The Johns Hopkins University School of Medicine, Baltimore, MD, United States

**Keywords:** biomimetic nanoparticles, cancer cell membranes, theranostics, immune response, metastasis

## Abstract

Nanoparticles (NPs) camouflaged in cell membranes represent novel biomimetic platforms that can mimic some of the membrane functions of the cells from which these membranes are derived, in biological systems. Studies using cell membrane coated NPs cover a large repertoire of membranes derived from cells such as red blood cells, immune cells, macrophages, and cancer cells. Cancer cell membrane coated nanoparticles (CCMCNPs) typically consist of a NP core with a cancer cell plasma membrane coat that can carry tumor-specific receptors and antigens for cancer targeting. The NP core can serve as a vehicle to carry imaging and therapeutic moieties. As a result, these CCMCNPs are being investigated for multiple purposes including cancer theranostics. Here we have discussed the key steps and major issues in the synthesis and characterization of CCMCNPs. We have highlighted the homologous binding mechanisms of CCMCNPs that are being investigated for cancer targeting, and have presented our data that identify BT474 CCMCNPs as binding to multiple cancer cell lines. Current preclinical applications of CCMCNPs for cancer theranostics and their advantages and limitations are discussed.

## Introduction

Biomimetic nanoparticles (NPs) are an emerging class of NPs that integrate the functionality of biological materials with the flexibility of synthetic materials to achieve effective navigation and interfacing in complex biological systems (1). Cell membrane coated NPs are biomimetic platforms that combine the functionality of cell membranes with the abilities of synthetic core structures to carry imaging reporters and therapeutic cargo (2).

Red blood cell (RBC) membrane-camouflaged poly(lactic-co-glycolic acid) (PLGA) NPs are one of the first reported cell membrane coated NPs. The RBC membrane was found to act as a nanosponge for toxins, and bestowed a longer circulation pharmacokinetic profile than uncoated NPs (3). Since this initial study, cell membrane coating technology has significantly expanded to the use of membranes from platelets (4–8) and from nucleated cells, such as macrophages (9–12), neutrophils (13), beta cells (14), and cancer cells (15–33). The use of cancer cell plasma membranes has attracted attention because these membranes carry tumor-specific receptors and antigens that play a role in cancer cell proliferation, invasion, and metastasis. While several comprehensive reviews have summarized the applications of cell membrane camouflaged NPs (34–46) from various cell sources, to the best of our knowledge only one of these reviews is focused on advances with CCMCNPs (37).

Here, we have discussed the steps and issues in the manufacturing and characterization of CCMCNPs. We have highlighted the homologous binding ability of CCMCNPs with potential applications in cancer targeting, and summarized the current preclinical applications of CCMCNPs for cancer theranostics, and their advantages and limitations.

## Synthesis of CCMCNPs

**Figure 1:**
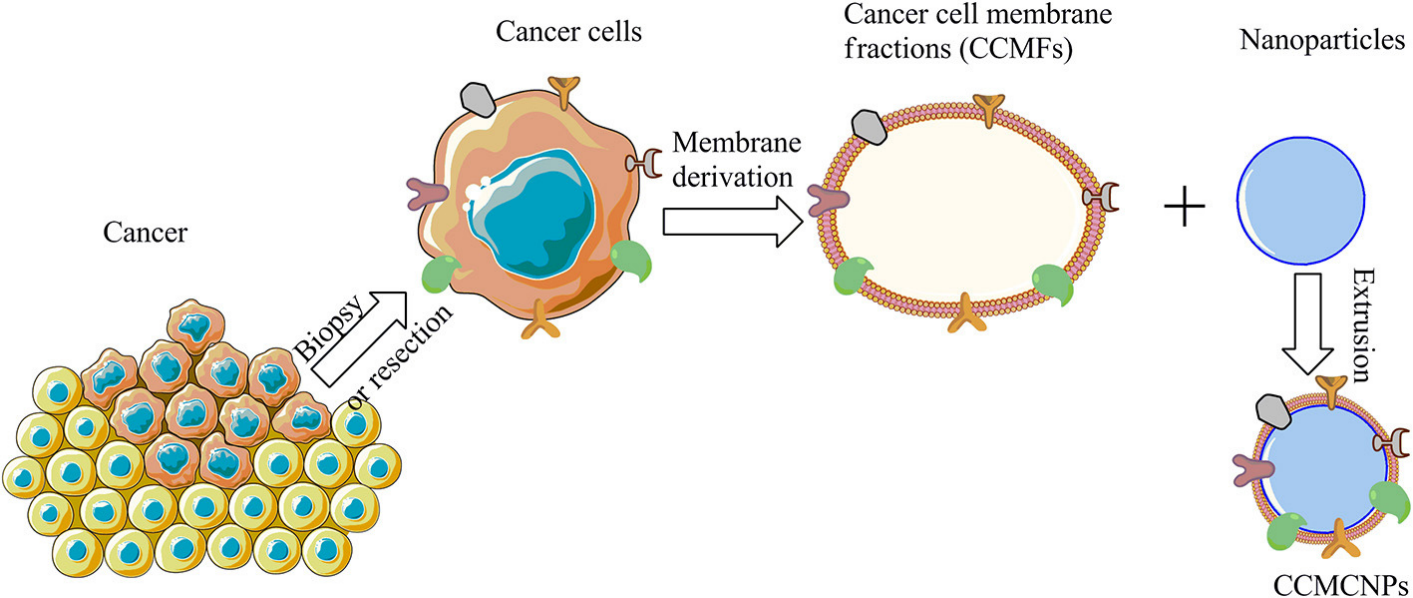
Schematic illustration of the steps required in the synthesis of CCMCNPs. The schematic was produced, in part, by using the graphics from powerpoint, ChemBioDraw and Servier Medical Art image data bank (https://smart.servier.com).

**Figure 2:**
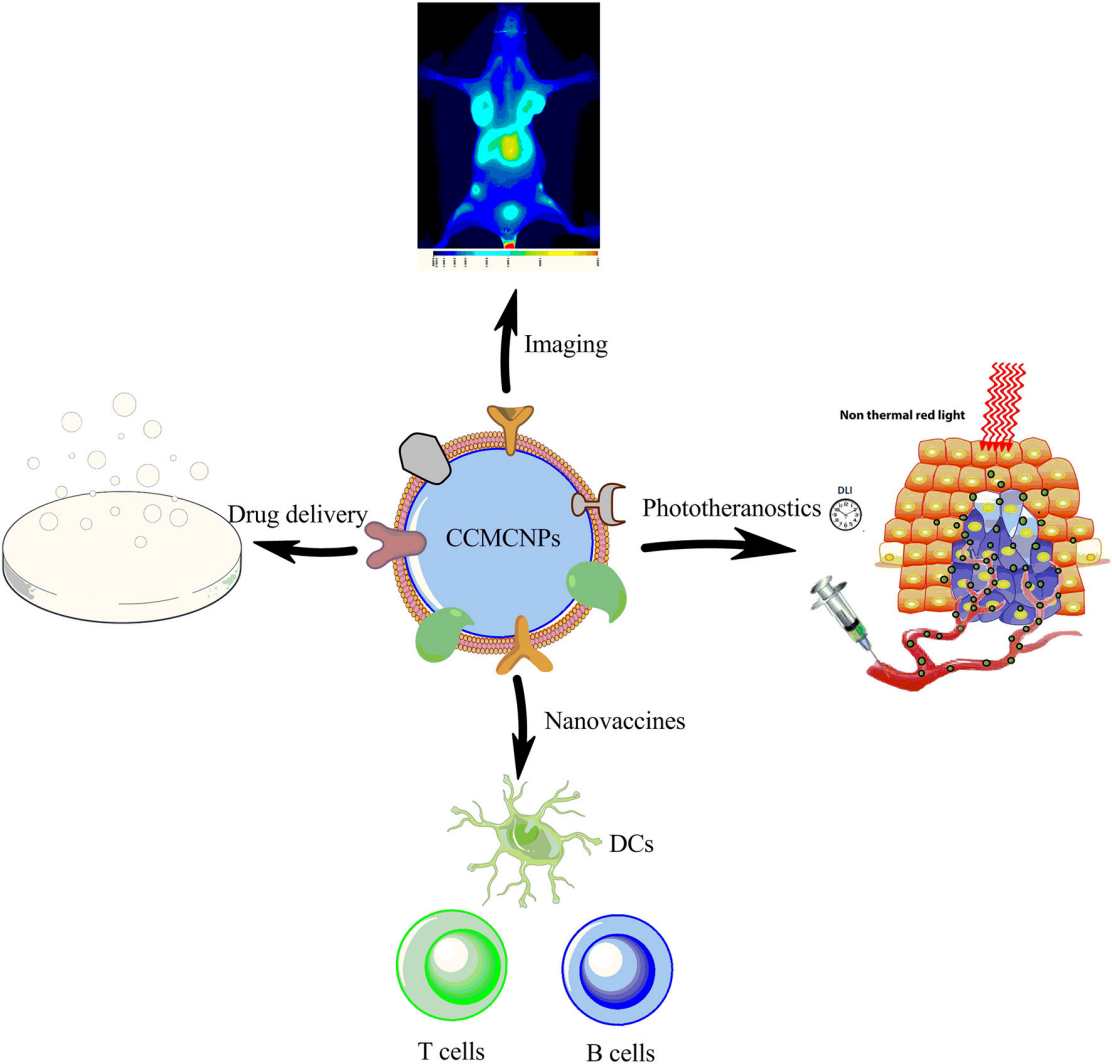
Schematic illustration summarizing the current applications of CCMCNPs in preclinical cancer theranostics. The schematic was produced, in part, by using the graphics from powerpoint, ChemBioDraw and Servier Medical Art image data bank (https://smart.servier.com).

CCMCNPs are synthesized by coating NPs with a lipid bilayer of cancer cell plasma membranes. The nanoparticle cores are synthetic materials that feature the advantage of flexibility and reproducibility. These cores can be used to carry therapeutic or imaging moieties. The cancer cell plasma membrane coating is a natural entity possessing the complexity and functionality derived from the cell membranes of the source cancer cells. As a result, CCMCNPs combine the advantages of synthetic and biological materials within a single biomimetic platform. A schematic illustration of the steps involved in the preparation of CCMCNPs is shown in Figure 1. Typical applications of CCMCNPs for cancer theranostics are shown in the schematic in Figure 2.

### CCMCNP Core Materials

The core of the CCMCNPs can be organic or inorganic. Organic nanoparticle cores include PLGA (15–17, 20, 31), semiconducting polymers (23), or poly(caprolactone) (PCL)-pluronic copolymers F68 (27). Polymeric NPs are usually biocompatible and biodegradable, and are used to encapsulate both hydrophilic and hydrophobic drugs for drug delivery. In particular, PLGA, an FDA approved polymer, has been widely investigated for pharmaceutical formulation applications (47). The polymer cores with their payloads are usually prepared via nano-precipitation followed by emulsion solvent evaporation, or self-assembly methods. Inorganic core materials used for CCMCNPs include silicon/silica (18, 30, 32), magnetic materials (22, 33), copper sulfide (CuS) (29), upconversion NPs (26), gold nanoshells (28), and metal–organic framework (MOF) (24, 25). Inorganic NP cores can exist in a porous structure to improve the drug loading efficiency and feature several unique electrical, magnetic, and optical properties adapted to meet to the specific need of the biomedical application. With recent advances in nanotechnology, inorganic NPs can be prepared with precise control of shape, size, and surface chemistry.

### Cancer Cell Membranes

Unlike cancer cells that have a nucleus and organelles, RBCs do not have a nucleus and lack organelles. As a result, RBC membrane vesicles are relatively easily obtained by removing the hemoglobin content under hypotonic conditions. The isolation of cancer cell membranes is, however, a more complex process. Typically, cancer cells are first ruptured into cell fragments under hypotonic conditions and with the use of mechanical processes, such as homogenization or sonication. Unbroken cells and nuclei are pelleted under low-speed centrifugation. The supernatant contains the plasma membrane, the cytosol and other organelles, such as lysosomes, golgi, mitochondria, and endoplasmic reticulum. Based on the density difference between the plasma membrane and other cellular factions, differential centrifugation is mainly used for plasma membrane isolation. However, we found that gradient centrifugation was superior to differential centrifugation in terms of purity of isolated cell membrane fractions (19). Commonly used gradients include discontinuous sucrose, and self-generated Percoll or iodixanol gradients (48, 49). In our recent study (19), we employed sucrose gradient centrifugation and demonstrated a discernable separation of the plasma membrane portion at the top band that was clearly separated from two other discrete bands.

Plasma membrane protein isolation kits have also been used to isolate cancer cell plasma membranes (23), with various commercial available kits from different vendors. However, one major concern with these commercial kits is that they are designed to isolate plasma membrane proteins but not the intact phospholipid vesicles to which the membrane proteins are attached or embedded. As a result the plasma membrane vesicles are frequently too small (around 50 nm) and have a poor yield.

### Cancer Cell Membrane Coating on NPs

Cancer cell membrane coating is commonly achieved though sonication and extrusion processes after mixing cancer cell membrane vesicles and NPs. Sonication is a pre-treatment process to break down and disperse cancer cell membrane vesicles. In an extrusion approach, a mini extruder used to make liposomes is connected to two air-tight syringes. Cancer cell membrane vesicles are mixed with NPs at a certain ratio and placed in the syringe on one side. Mechanical force is applied on the plunger of the syringe, and the mixture is extruded through a polycarbonate porous membrane located in the center of extruder. Cancer cell membrane coated NPs are collected after several passes through the polycarbonate membrane.

The mechanical force applied while the membrane vesicles and NPs are passing through the small pores of the polycarbonate membrane facilitates the fusion between vesicles and nanoparticle. This extrusion-based preparation method is adapted from liposome synthesis technology. However, this method suffers from several limitations when it is applied to fabricate CCMCNPs. Cancer cell membrane vesicles produced by mechanical rupture appear in a variety of sizes and shapes. Because they are much thicker and more rigid than liposomes, this hampers the curving of membrane vesicles for nanoparticle coating, and also results in pore obstruction of the polycarbonate membrane and loss of membrane vesicles. Due to these technical difficulties, the coating efficiency is largely compromised. The resulting product usually contains some NPs without membrane coating, and some membrane vesicles without an NP core. Unfortunately, because there is no efficient method to remove the uncoated NPs or the membrane vesicles, the final product contains a small fraction of both these unwanted components. In addition, this laboratory-based small scale synthesis involves multiple manual steps that impede its clinical translation due to concerns such as synthesis variability and manufacturing scalability. Although the current extrusion-based coating method works for a variety of cell membranes and NPs, the coating mechanism and synthesis procedure require optimization. Such optimization should take into account the physicochemical properties of NPs, such as the size, shape, morphology, and surface chemistry that will impact coating efficiency.

Microfluidics provides a robust tool to produce nanomaterials in a controlled and reproducible manner. In a recent study, a microfluidic chip that incorporated electroporation was fabricated (50). Magnetic nanoparticles (MNs) and RBC membrane-derived vesicles (RBC-vesicles) were made to flow through the electroporation zone. Electric pulses between two electrodes promoted the entry of MNs into RBC-vesicles facilitating the synthesis of RBC-MNs. RBC-MNPs synthesized by the microfluidic electroporation approach exhibited significantly better treatment outcome than those prepared by conventional methods. Although not previously reported, such a microfluidic approach may provide a promising alternative to producing CCMCNPs.

Using cancer cell membranes to coat NPs is a top-down approach. Although it has the above-mentioned limitations in fabrication, it does recapitulate some of the biological complexities of the cell membrane on the carrier surface and provides a one-step solution to transferring some of the bioactive functions of the cell membrane to the carrier (51). Recently, a combined top down and bottom-up strategy was described that incorporated proteins derived from the leukocyte plasma membrane into lipid nanoparticles (51–53). The top-down strategy was used to obtain cell membrane proteins that were inserted into a lipid bilayer by a bottom-up approach. The resulting proteolipid vesicles, referred to as leukosomes, retained the versatility and physicochemical properties typical of liposomal formulations. This biomimetic approach provided better control of the final composition and formulation. However, it did not reproduce the complexity of the cellular membrane or maintain cell membrane protein conformation, surface density, or ratio.

## Characterization OF CCMCNPs

### Characterization of Physicochemical Properties

Transmission electron microscopy (TEM) is an effective way to characterize the size and morphology of CCMCNPs and their precursors. Cancer cell membrane vesicles exist in a coil-like shape with a broad size distribution. Core-shell structures observed in TEM images provide important evidence of successful coating. The thickness of the membrane coating ranges from 5 to 10 nm that is in agreement with the thickness of the phospholipid bilayer. Dynamic laser scattering (DLS) is another commonly used technique to detect the hydrodynamic size and zeta-potential of NPs. The hydrodynamic size of CCMCNPs is slightly higher than the size of the core NPs due to membrane coating, and much lower than cancer cell membrane vesicles. The zeta-potential of CCMCNPs is similar to that of membrane vesicles. Combined data from TEM and DLS can be used to confirm successful membrane coating. The stability of CCMCNPs can be evaluated by measuring the hydrodynamic size over a period of time in suspension in culture medium, serum or physiological buffers. The size fluctuation of CCMCNPs is minimal. Studies evaluating coagulation have not been reported so far.

### Protein Profiling by SDS-PAGE

A major goal of cancer cell plasma membrane coating is to translocate membrane proteins in an intact state on to the core NPs. It is therefore important to confirm the preservation of membrane proteins and their associated structures and functions, once the membrane is coated on the NP. SDS-PAGE is usually used to demonstrate the preservation of membrane proteins by confirming the unchanged protein profiles of cancer cell membrane vesicles before and after coating. However, the protein staining after SDS-PAGE is non-specific and all the proteins are stained. The technique is a relatively coarse approach to profile proteins and is not sensitive to protein changes. We addressed this issue by using membranes from cells overexpressing the G protein-coupled receptor CXCR4 and the glycoprotein CD44 (19), and confirming the levels of these markers before and after cell membrane coating, using flow cytometry.

### Purity, Integrity, and Sidedness

The purity of cancer cell plasma membrane vesicles is typically evaluated by western blot probing of a series of subcellular fractions along with purified membrane. We carefully examined the purity of the isolated membrane vesicles by probing a series of subcellular markers, Na^+^/K^+^-ATPase for plasma membrane, ATP5a for mitochondrial, GAPDH for cytosolic protein, and GRP78 for endoplasmic reticulum (19). We confirmed the successful enrichment of plasma membrane associated proteins and negligible contamination from subcellular organelle proteins. To check the purity of CCMCNPs, the core and shell are labeled with fluorescence dyes of different colors, and the overlapping of the two fluorescence signals is used to confirm that the cell membranes have coated the NPs. However, due to the limited spatial resolution of fluorescence microscopy, subtle mismatches at the nanoscale level can be missed by fluorescence microscopy.

To determine if CCMCNPs remain intact under physiological conditions, the most common approach is to label the NP core and the outer coat shell with different fluorescence dyes and check the fluorescence of CCMCNPs following internalization by cells. Although this approach is also subject to the limited spatial resolution of fluorescence microscopy, it is considered the simplest way to evaluate CCMCNP integrity.

As discussed earlier, sidedness or the orientation of the cancer cell plasma membrane is an important characteristic of CCMCNPs. The membrane coating on CCMCNPs has to be in a “right-side-out” manner for applications that require the extracellular domains of membrane proteins to be exposed, such as for antigen binding and recognition. The sidedness of RBC membranes after coating has been evaluated by using immunogold labeling (54), where antigen markers located at extracellular and intracellular domains were separately stained with colloidal gold NPs labeled with respective specific antibodies. However, the sidedness of CCMCNPs has not been well-investigated, and immunogold labeling combined with electron microscopy will be useful techniques for evaluating CCMCNP sidedness. Although electron microscopy of immunogold labeling can determine membrane sidedness at high spatial resolution, flow cytometry is better adapted to quantify translocated membrane proteins (51).

## Targeting Mechanisms OF CCMCNPs

### Homologous Binding of CCMCNPs to Cancer Cells

Cell adhesion molecules, including cadherins, selectins, integrins, and Thomsen–Friedenreich (TF) antigens, have been identified as mediators of cell-cell and cell-extracellular matrix adhesion (55). These play a pivotal role in recurrence, invasion, and distant metastasis (55). Homotypic aggregation and metastatic cell heterotypic adhesion to the microvascular endothelium are thought to be mediated through similar molecular mechanisms, specifically the interactions of tumor-associated TF glycoantigen with the beta-galactoside-binding protein, galectin-3 (56, 57). The homologous targeting ability of CCMCNPs was first evaluated using PLGA NPs coated with the plasma membrane of human MDA-MB-435 cancer cells (17). In these studies, CCMCNPs had a much higher affinity toward MDA-MB-435 cells compared to RBC-camouflaged NPs and bare PLGA cores. When a heterotypic human foreskin fibroblast cell line was used as a negative control, MDA-MB-435 CCMCNPs exhibited little increased uptake compared to bare PLGA cores, indicating that the homologous binding effect was specific to the MDA-MB-435 cell membrane coating.

Following these pioneering studies, several published reports have observed homologous binding of CCMCNPs derived from a variety of cancer cells, such as mouse breast cancer 4T1 cells (24, 25, 27, 28), MDA-MB-435 cells (26), human breast cancer MDA-MB-231 (15), and MCF-7 (16) cells, human squamous carcinoma UM-SCC-7 cells (33), and human hepatocellular carcinoma SMMC-7721 cells (22). CCMCNPs derived from these cancer cells demonstrated homologous specificity in preclinical cancer imaging or therapy studies.

**Figure 3:**
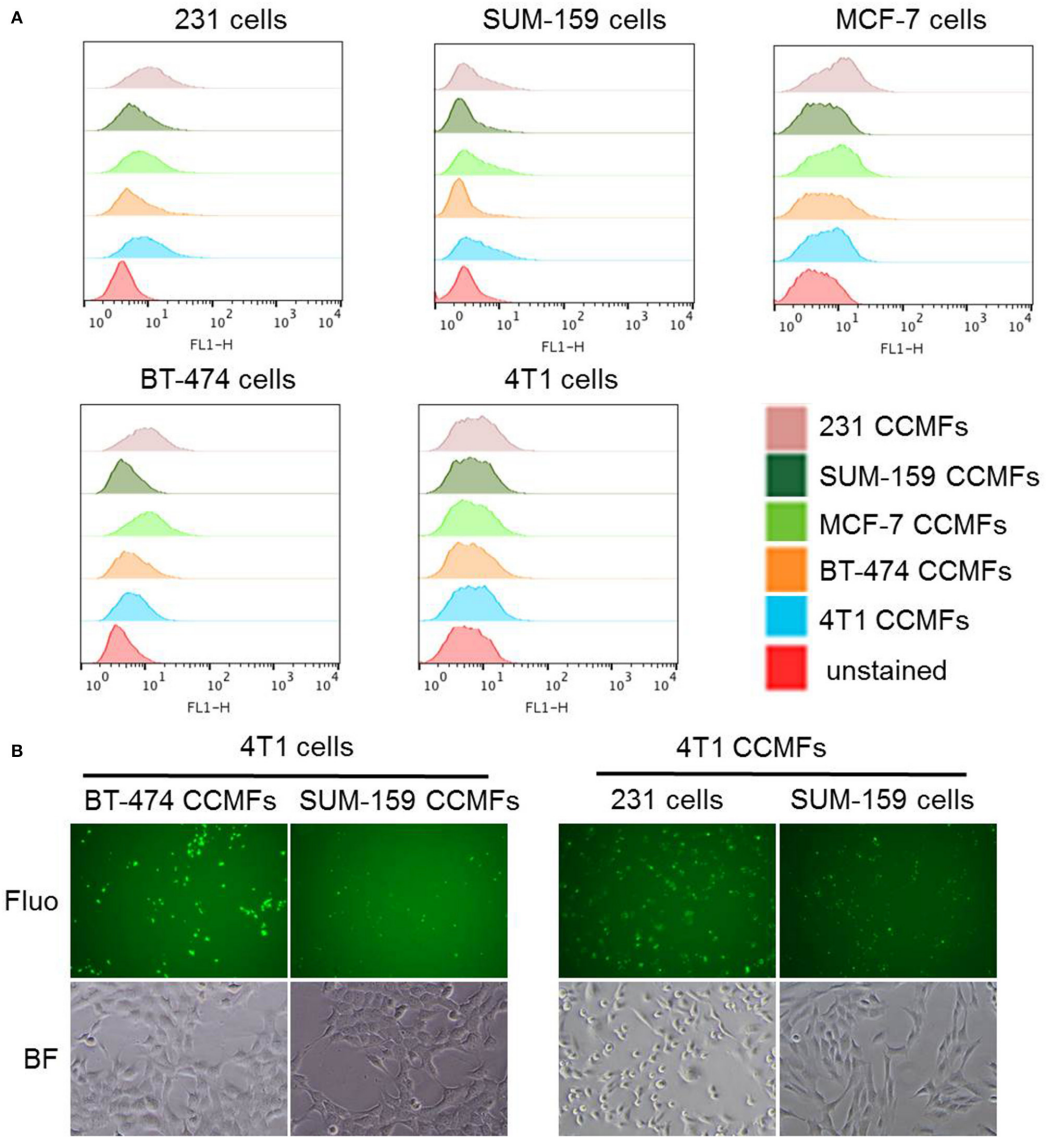
Evaluation of homologous binding by flow cytometry and fluorescence imaging. **(A)** Flow cytometry profiles of five breast cancer cell lines, MDA-MB-231, SUM-159, MCF-7, BT-474, and 4T1 cells after CCMF-incubation. Live cells were individually stained with FITC-labeled CCMFs derived from these five cell lines at the same FITC concentration. **(B)** Representative fluorescence (Fluo) and bright field (BF) images of cancer cells after CCMF-incubation showing the high binding affinity of BT-474 CCMFs and MDA-MB-231 cells, compared to the low binding affinity of SUM-159 CCMFs and SUM-159 cells.

We performed a study in which membranes derived from five breast cancer cell lines (MDA-MB-231, SUM-159, MCF-7, BT-474, and 4T1) were evaluated for homologous binding toward their source cell lines. Cancer cell membrane fractions (CCMFs) were first fluorescently labeled with fluorescein isothiocyanate (FITC). FITC-labeled CCMFs were characterized by measuring the absorbance at 494 nm by UV spectroscopy. 1 × 10^6^ live cells were dispersed in 100 μL of FACS buffer, after which 5 μL of FITC-labeled CCMF solution (A_494_ = 0.4) was added and the mixture was incubated at 4°C for 30 min. After washing, flow cytometry measurements were conducted on a FACS Calibur (BD Bioscience); ten thousand events were collected for each measurement and analyzed by FlowJo software (BD Bioscience). In a separate study, live cells were incubated with FITC-labeled CCMFs at 37°C for 1 h. After washing, the live cells were imaged by fluorescence microscopy. As shown in Figure 3A and Table 1, although we did not see homologous binding of the CCMFs investigated, we found that BT474 CCMFs had a higher affinity for all five cell lines, suggesting that BT474 cell membranes showed promiscuous binding to these five cancer cell lines. MDA-MB-231 cells had the highest binding affinity for all the CCMFs. SUM-159 CCMFs and SUM-159 cells had the lowest binding affinity. Representative fluorescence images shown in Figure 3B confirm that MDA-MB-231 cells had the highest level of CCMF binding among the five cell lines that was consistent with the flow cytometry results. The fluorescent images confirm the low binding affinity of SUM-159 CCMFs and cells. These results support further investigating the use of NPs coated with BT474 cancer cell membranes to detect cancers and to deliver therapeutic cargo.

**Table 1:** Flow cytometry analysis of mean fluorescence intensity (MFI) obtained following incubation of different CCMFs with different cancer cells.

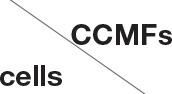	**MDA-MB-231**	**SUM-159^**c**^**	**MCF-7**	**BT-474^**a**^**	**4T1**
MDA-MB-231^b^	14.1	8.8	17.3	32.8	13.5
SUM-159^c^	6.4	4.2	7.3	7.8	7.8
MCF-7	14.2	7.1	12.0	22.3	10.7
BT-474	12.8	5.9	14.6	14.0	8.7
4T1	12.1	10.8	13.1	25.0	10.4

*Values are obtained from two separate sets of experiments*.

a *BT474 CCMFs had a higher binding affinity than other CCMFs across the five cell lines as outlined by a vertical box*.

b *From the five cell lines, MDA-MB-231 cells had the highest binding affinity as outlined by a horizontal box*.

c *SUM-159 CCMFs and SUM-159 cells had the lowest binding affinity*.

Other studies have reported an absence of homologous binding *in vivo*. In one such study, semiconducting polymer nanoparticles (SPN) were coated with the plasma membrane derived from 4T1 cells to form 4T1 CC-SPN (23). The core SPN absorbed near-infrared (NIR) light that was converted to NIR fluorescence and photoacoustic signals. In addition, SPN can be also used for photodynamic therapy (PDT) and photothermal therapy (PTT). Homologous targeting of 4T1 CC-SPN was evaluated in 4T1 tumors. Compared to naked SPN, 4T1 CC-SPN did not show significant enhancement of fluorescence or photoacoustic intensities in 4T1 tumors, indicating that the accumulation of 4T1 CC-SPN in 4T1 tumors was not significantly different from naked SPN. Following phototherapy, the 4T1 CC-SPN group did not show significant difference of tumor growth control in comparison to the naked SPN group.

Many of the homologous targeting studies used uncoated NPs for comparison. If the homologous targeting of CCMCNPs originates from cell surface adhesion molecules, studies with specific inhibitors with competitive binding to these adhesion molecules will provide further insights into the mechanisms underlying the homotypic and heterotypic effects.

Several studies have suggested that the CD47 cell surface antigen may suppress the uptake of CCMCNPs by macrophages (27). On the other hand, neoantigens encoded by tumor-specific mutated genes can help elicit tumor-specific immune responses (58). We investigated the pharmacokinetic profiles of CCMF-PLGA NPs and uncoated PLGA NPs in immunodeficient mice and found that CCMF-PLGA NPs had even significantly shorter circulation time in the bloodstream, indicating that the membrane antigens present on CCMF-PLGA NPs may have accelerated clearance (19). These opposing effects require further investigation to clearly understand how CCMCNPs interface with mononuclear phagocytic system.

### Disruption of Cancer Cell-Stromal Cell Interaction

Proteins present on the membranes of source cells are maintained on the surface of CCMCNPs. Some of these proteins play a role in mediating cell-cell interaction. We therefore investigated the ability of CCMCNPs to misdirect cancer cell-stromal cell interaction (19). We found that the presence of CCMFs or CCMCNPs reduced the migration of cancer cells toward human mammary fibroblasts (HMFs) by ~30%. Core PLGA NPs alone did not induce any inhibition of cancer cell migration. We established an experimental metastasis model by intravenously injecting MDA-MD-231 cells constitutively expressing luciferase (231-luc) into nude mice. We observed a significant reduction of metastasis in the group of mice injected with 231-luc cells pre-incubated with CCMCNPs. Our data collectively showed that CCMCNPs actively reduced the ability of fibroblasts to attract cancer cells, and confirmed the ability of CCMCNPs to significantly reduce metastasis. Fibroblasts have been observed to track to the premetastatic niche prior to the arrival of cancer cells. Therefore, NPs that disrupt the ability of fibroblasts to attract cancer cells may disrupt the metastatic cascade and the formation of metastasis. However, we have not as yet identified the specific membrane antigens responsible for the reduction of migration.

## Cancer Theranostic Applications OF CCMCNPs

Since the NP cores are synthetic materials, these can be adapted for use as theranostic materials to serve as vehicles that carry imaging and therapeutic moieties. We have summarized the reported examples of CCMCNPs for cancer theranostics in Table 2.

**Table 2:** Summary of CCMCNPs and their components used for cancer theranostics.

**Cancer cell line**	**Core**	**Size (nm)**	**Imaging/therapeutics**	**Applications**	**References**
MDA-MB-435	UCNPs	100	UCNPs	NIR imaging	(26)
MDA-MB-831	mPEG-PLGA	70	IR780	NIR imaging	(21)
MCF-7	PLGA	200	ICG	NIR/PA imaging and PTT	(16)
SMCC-7721	SPIO	192	Ce6	MR/NIR imaging and PDT	(22)
4T1	PCN-224	228	GOx and catalase	Cancer starvation and PDT	(25)
4T1	PCN-224	154	TPZ	Bioreductive therapy and PDT	(24)
4T1	F68 copolymer	175	PTX	Drug delivery	(27)
UM-SCC-7	MNPs	103	DOX	Drug delivery	(33)
4T1	Gold nanocages	70	DOX	Drug delivery and PTT	(28)
B16-F10 & RBC	Copper sulfide	200	DOX	Drug delivery and PTT	(29)
MDA-MB-231	Porous Silicon	405	N/A	Cancer nanovaccines	(18)
B16-F10	PLGA	110	CpG	Cancer nanovaccines	(20)
B16-F10	PLGA	160	R837 and mannose	Cancer nanovaccines	(31)

*UCNPs, upcoversion nanoparticles; NIR, near infrared; PLGA, poly(lactic-co-glycolic acid); SPIO, superparamagnetic iron oxide; MR, magnetic resonance; PA, photoacoustic; PDT, photodynamic therapy; PTT, photothermal therapy; MNPs, magnetic nanoparticles; PTX, paclitaxel; DOX, doxorubicin; GOx, glucose oxidase; PCN, porous coordination network, TPZ, tirapazamine*.

### Preclinical Cancer Imaging

Cloaking cancer cell membranes onto upconversion nanoparticles (UCNPs) to obtain CC-UCNPs have been reported (26). Four cancer cell lines including human melanoma, prostate, squamous cell, and colorectal cells were selected to synthesize the corresponding CC-UCNPs. The homologous binding of CC-UCNPs was investigated *in vitro* by flow cytometry and confocal microscopy. Significant binding was observed when the cell membrane of the CC-UCNPs matched the cancer cell type. Mismatch between the donor and host cells led to almost no targeting. By virtue of the UCNP core's ability to convert NIR radiation to visible light, CC-UCNPs possessed the ability for *in vivo* tumor imaging. Mice injected with CC-UCNPs derived from MDA-MB-435 cells exhibited the highest upconversion luminescence in MDA-MB-435 tumor xenografts, as well as much higher tumor accumulation than the CC-UCNPs from other cell lines. These homologous targeting abilities together with the NIR fluorescence of UCNPs indicate the potential use of CC-UCNPs for tumor specific *in vivo* imaging.

In another study, a brain metastatic breast cancer cell (MDA-MB-831) membrane-coated polymeric nanoparticle (mPEG-PLGA) platform was constructed (21). NIR dye IR780 was loaded into the mPEG-PLGA polymeric NPs for imaging. *In vivo* and *ex vivo* NIR imaging in mice showed extended circulation and retention of MDA-MB-831 CCMCNPs compared to uncoated mPEG-PLGA nanoparticles. These data demonstrated the ability of dye-loaded CCMCNPs to cross the blood-brain barrier (BBB) for imaging of metastatic breast cancers to the brain. These two examples represent applications of CCMCNPs for NIR tumor imaging, where the NIR light is able to penetrate deeper into the tissue than visible light. Although the penetration of NIR light makes superficial tumor imaging possible, it cannot be applied to deep-seated tissues. Magnetic nanoparticles are an alternative option as they allow detection of deep-seated tissues with MRI, and pave the way for translational applications. To be clinically translatable, cancer cell membranes can also be labeled with radiotracers for detection *in vivo* by PET/SPECT imaging.

### Phototheranostics

A cancer cell membrane–cloaked NP as a phototheranostic nanoplatform has been previously reported (16). The NP core consisted of PLGA containing indocyanine green (ICG) that has excellent fluorescence/photoacoustic (FL/PA) properties for FL/PA dual-modal imaging and PTT effects for eradicating tumors using NIR light. The membranes of human breast cancer MCF-7 cells were used for coating. MCF-7 CCMCNPs not only demonstrated homologous targeting *in vitro* but also demonstrated specific targeting with *in vivo* MCF-7 tumors with high spatial resolution and good penetration. Due to the PTT effect, MCF-7 tumors were ablated with a single dose of MCF-7 CCMCNPs combined with laser treatment.

In another study, a cancer cell membrane coated magnetic NP platform for MR/NIR fluorescence dual-modal imaging and PDT of cancer was described (22), where the core consisted of styrene (St) and acrylic acid (AA)-crosslinked superparamagnetic iron oxide nanoparticles (SPION), loaded with a clinically used photosensitizer Ce6. The nanobead core was coated with the membranes from human hepatocellular carcinoma SMMC-7721 cells. Compared to nanobeads without coating, SMMC-7721 CCMCNPs demonstrated higher tumor accumulation as observed by MR/NIR fluorescence imaging, and enhanced PDT effects in SMMC-7721 tumor-bearing mice.

In two recent studies, cancer cell membrane camouflaged cascade bioreactors (designated as mCGP) were used for a synergistic combination of starvation and PDT (24, 25). The core consisted of porphyrin MOF loaded with glucose oxidase (GOx) and catalase. PCN (porous coordination network)-224 acted as a photosensitizer and also had photoluminescence suitable for NIR imaging. Coating the surface with 4T1 cancer cell membranes provided mCGP with biocompatibility, immune system-evasion and homotypic targeting. Once internalized by cancer cells, mCGP promoted microenvironmental oxygenation by catalyzing the endogenous H_2_O_2_ to produce O_2_ that subsequently accelerate the decomposition of intracellular glucose and enhanced the production of cytotoxic singlet oxygen under light irradiation. This cancer targeted cascade bioreactor mCGP efficiently inhibited cancer growth after administration of a single dose.

As highlighted in the examples presented here, the integration of imaging with phototherapy enabled real-time *in vivo* monitoring of the distribution of CCMCNPs to identify the ideal time to trigger treatment for an optimal therapeutic effect.

### Chemotherapy Drug Delivery

CCMCNPs can be effective drug delivery nanocarriers when the NP cores are loaded with chemotherapy payloads as demonstrated in published studies. In one study, a cancer cell biomimetic nano drug delivery system (NDDS) was developed for targeted chemotherapy of metastatic cancer (27). The NDDS was constructed from two distinct components. The NP coat derived from the membranes of 4T1 mammary breast cancer cells formed one component. The second component consisted of the paclitaxel (PTX)-loaded polymeric NP core prepared from poly(caprolactone) (PCL) and pluronic copolymer F68. The preservation of several membrane proteins associated with cell adhesion and recognition was confirmed. Among these were TF-antigen and E-cadherin, CD44 and CD326, and CD47. The 4T1 CCMCNPs could selectively enable high accumulation of PTX in primary tumors and metastatic pulmonary tissues as demonstrated in 4T1 orthotopic mammary tumors and experimental metastasis. The membrane proteins present on CCMCNPs were postulated to result in homotypic binding of CCMCNPs and in the reduction of internalization by macrophages. In addition, 4T1 CCMCNPs significantly inhibited the growth of primary and metastatic tumors.

Besides polymeric NP cores, several inorganic NPs have been used to encapsulate chemotherapy drugs. Examples are magnetic nanoparticles (33), gold nanocages (28), and CuS (29). As an example, a magnetic iron oxide based nanoplatform that was coated with cancer cell membranes from different cancer cells, such as UM-SCC-7, COS7, and HeLa cells was recently described (33). Clinically used doxorubicin hydrochloride (DOX·HCl) was incorporated as the model drug that electrostatically bound to the negatively charged Fe_3_O_4_ magnetic NPs. These CCMCNPs exhibited excellent “homing” ability to the homologous tumor *in vivo* even in the presence of a second heterologous tumor. Due to the targeted accumulation of DOX in UM-SCC-7 tumors, UM-SCC-7 CCMCNPs resulted in significant tumor growth control. The magnetic property of the NP core also allowed the use of a magnetic field to guide CCMCNPs toward the site of interest to increase accumulation. In another study, a biomimetic drug delivery system consisting of DOX-loaded gold nanocages as the inner core and 4T1 cancer cell membranes as the outer shell was used for homologous targeting to 4T1 tumors, with the gold nanocages providing both PTT effects and hyperthermia-triggered DOX release under NIR laser irradiation (28). The combination of chemo and PTT therapy achieved about 98% growth reduction of primary and metastatic 4T1 tumors. Hollow CuS NPs have also been used to load DOX (29). Similar to the gold nanocages, DOX-loaded CuS NPs provided PTT effects and NIR light-triggered DOX release. Instead of using cancer cell membrane alone, in these studies membranes derived from RBCs and melanoma cells (B16-F10 cells) were fused to create hybrid biomimetic membranes (RBC-B16) that were coated onto DOX-loaded CuS NPs to construct DCuS@[RBC-B16] NPs. The hybrid membrane coating provided functional advantages of homologous targeting from the B16-F10 cell membranes and prolonged circulation time from the RBC membranes. Compared to the bare CuS NPs, the DCuS@[RBC-B16] NPs exhibited specific self-recognition to the source cell line *in vitro* and achieved prolonged circulation lifetime and enhanced homogeneous targeting abilities. The DOX-loaded [RBC-B16]-coated CuS NP platform exhibited synergistic PTT and chemotherapy to achieve significant tumor growth inhibition.

### Cancer Nanovaccines

Because cancer cell membranes carry a repertoire of membrane proteins from their source cancer cells, CCMCNPs have been actively investigated as cancer nanovaccines to induce cancer-specific immune responses. Subunit vaccines, such as molecular adjuvants and cancer-associated antigens or cancer-specific neoantigens, have been demonstrated to elicit potent antitumor immunity (59). However, subunit vaccines have shown limited clinical benefit in cancer therapy, due in part to inefficient vaccine delivery. Nanovaccines possess several favorable characteristics for effective cancer immunotherapy, such as efficient co-delivery of antigens and adjuvants into lymphoid organs, controllable intracellular vaccine delivery and release, and antigen cross-presentation in antigen presenting cells (APCs). The earliest example of using CCMCNPs to induce anti-tumor immunity *in vivo* was reported by Kroll et al. (20). The anticancer nanovaccine contained a PLGA polymeric core loaded with CpG oligodeoxynucleotide 1826 (CpG), a nucleic acid-based immunological adjuvant, and a cancer cell membrane shell derived from B16-F10 mouse melanoma cells. The nanovaccine resulted in a potent antitumor immune response *in vivo* and exhibited substantial therapeutic effects when combined with additional immunotherapies such as immune checkpoint blockades.

In a similar study (31), with a nanovaccine containing a PLGA core and a B16-F10 cell membrane shell, the toll-like receptor 7 agonist imiquimod (R837) was used as an adjuvant instead of CpG. Additionally the surface was modified with mannose as an APC-recognition moiety. This modified nanovaccine demonstrated both prophylactic and therapeutic efficacy *in vivo*, and enhanced therapeutic effects when combined with anti-PD-1 immune checkpoint blockade therapy.

We performed a proof-of-principle study investigating the immune response induced by CCMCNPs (19). We used human glioblastoma cells (U87MG) and human breast cancer cells (MDA-MB-231 and BT-474) for the derivation of cell membranes. We observed the localization of CCMCNPs in proximal draining lymph nodes with NIR fluorescence imaging. Following immunization of Balb/c mice, we detected a higher percentage of CD8^+^ and CD4^+^ cytotoxic T-lymphocyte populations in spleens and lymph nodes of CCMCNPs-immunized mice. Since U87-CXCR4 cells are of human origin and the CCMCNPs were injected into immunocompetent mice, our study did not demonstrate a cancer-cell specific immune response, but identified the possibility of using such formulations together with immunogenic adjuvants in combination with checkpoint inhibitors for cancer treatment.

CCMCNPs-based nanovaccines provide several advantages for cancer immunotherapy. Although many intracellular housekeeping proteins are removed that can dilute the immune responses, the membrane-bound tumor antigens are retained creating the possibility of synthesizing personalized vaccines. Tumor antigens from CCMCNPs are multi-epitope and endogenously autologous that can potentially be derived from a patient's own tumor following surgical resection or biopsy. The core-shell structure of CCMCNPs allows the delivery of tumor antigens and adjuvant concurrently to maximize effective antigen presentation and activation of downstream immune processes (20).

However, such bioinspired nanovaccine platforms are still in their infancy. The prophylactic and therapeutic effects generated from CCMCNPs have to be optimized. Cancer cell membranes also contain housekeeping proteins that can result in immune response dilution. In addition, the antigens located in the cell membranes can be degraded or inactivated in the complex physiological environments of the host body. Finally, the potential adverse effects of immunomodulatory cocktails have to be considered.

## Concluding Remarks and Future Perspectives

Homologous targeting to deliver imaging and therapeutic agents, disruption of cancer cell-stromal cell interactions, and induction of an immune response are the major cancer applications that are emerging with this class of NPs. Overall, this novel biomimetic nanoplatform allows the incorporation of personalized cancer receptors and antigens, which can, in the future, be derived from a patient's own tumor. Furthermore, the NP cores can be loaded with a variety of different cargoes, for precision medicine. The challenges that need to be addressed to enable translation to applications in humans are reproducible synthesis under good medical practice conditions that can be scaled up, and understanding the mechanisms underlying homologous targeting.

Current synthesis involves multiple manual steps that can introduce process variability. Some important characteristics, such as purity, integrity, and sidedness, in particular, need to be further investigated and elucidated. There are very few studies reporting the yield, loading capacity, or efficacy of CCMCNPs. Successfully addressing these challenges will allow the incorporation of this novel class of NPs for cancer theranostics to achieve personalized precision medicine.

## Author Contributions

JJ wrote the manuscript and performed the experiments. ZB wrote and edited the manuscript.

### Conflict of Interest

The authors declare that the research was conducted in the absence of any commercial or financial relationships that could be construed as a potential conflict of interest.
